# Harnessing the Power of Venomous Animal-Derived Toxins against COVID-19

**DOI:** 10.3390/toxins15020159

**Published:** 2023-02-14

**Authors:** Isadora Oliveira, Isabela Ferreira, Beatriz Jacob, Kiara Cardenas, Felipe Cerni, Djane Baia-da-Silva, Eliane Arantes, Wuelton Monteiro, Manuela Pucca

**Affiliations:** 1 Department of BioMolecular Sciences, School of Pharmaceutical Sciences of Ribeirão Preto, University of São Paulo, Ribeirão Preto 14040-903, SP, Brazil; 2 Medical School, Federal University of Roraima, Boa Vista 69310-000, RR, Brazil; 3 Health Sciences Postgraduate Program, Federal University of Roraima, Boa Vista 69310-000, RR, Brazil; 4 Institute of Clinical Research Carlos Borborema, Dr. Heitor Vieira Dourado Tropical Medicine Foundation, Manaus 69850-000, AM, Brazil; 5 Postgraduate Program in Tropical Medicine, School of Health Sciences, Amazonas State University, Manaus 69850-000, AM, Brazil; 6 Department of Collective Health, Faculty of Medicine, Federal University of Amazonas, Manaus 69077-000, AM, Brazil; 7 Leônidas and Maria Deane Institute, Fiocruz Amazônia, Manaus 69057-070, AM, Brazil; 8 Faculty of Pharmacy, Nilton Lins University, Manaus 69058-040, AM, Brazil

**Keywords:** SARS-CoV2, COVID-19, coronavirus, animal toxins, venoms

## Abstract

Animal-derived venoms are complex mixtures of toxins triggering important biological effects during envenomings. Although venom-derived toxins are known for their potential of causing harm to victims, toxins can also act as pharmacological agents. During the COVID-19 pandemic, there was observed an increase in in-depth studies on antiviral agents, and since, to date, there has been no completely effective drug against the global disease. This review explores the crosstalk of animal toxins and COVID-19, aiming to map potential therapeutic agents derived from venoms (e.g., bees, snakes, scorpions, etc.) targeting COVID-19.

## 1. Introduction

A virus is a non-cellular agent with nucleic acid surrounded by a protein coating (some may also have a lipid envelope over the capsid) and which can only reproduce within host cells [1]. Virus-borne diseases affect millions of people every year, causing mild infections to severe complications and deaths, such as Human Immunodeficiency Virus/Acquired Immunodeficiency Syndrome (HIV-AIDS) and Coronavirus Disease 2019 (COVID-19) [2,3]. COVID-19 is an important cause of morbidity and mortality; however, the pathogenic mechanisms are still poorly understood, and possible treatments are being explored in a growing phase of studies [4].

Animal-derived venoms are rich and complex sources of bioactive compounds that can act as antiparasitic, antimicrobial, and antiviral agents [5,6,7]; indeed, some antimicrobial peptides (AMPs) from animal venoms have shown antiviral activity such as melittin, phospholipases (PLA_2_), and L-amino oxidases (LAAO), albeit they are usually cytotoxic to host cells [8]; thus, bioactive compounds, such as toxins, frequently need to be modified to make them more selective [9,10,11]. Unlike molecules that interact with virus lipids, molecules targeting specific virus proteins may interfere with the virus replication or affect the interaction between the virus and the host cell, making them potentially more selective; in addition, compared to traditional small molecule drugs, natural proteins and peptides exhibit higher specificity and potency to their targets [12].

This review presents COVID-19 in its historical, epidemiological, and pathological contexts, in addition to addressing the use of animal-derived toxins against the disease, providing a new approach to the subject and new perspectives of treatment.

## 2. COVID-19 Disease

In 2020, the world suffered a chaotic situation from the pandemic of COVID-19, a disease caused by the severe acute respiratory syndrome coronavirus 2 (SARS-CoV-2) which first emerged from China (December 2019) [13,14,15]. So far, it has been reported that more than 650 million people have been infected by the disease and there have been 6.7 million deaths [3]. The signs and symptoms of COVID-19 have been considered very complex, ranging from flu-like mild symptoms to a severe spectrum in high-risk individuals [16]; indeed, COVID-19 symptoms include common fever, cough, fatigue, slight dyspnea, sore throat, headache, conjunctivitis, and serious complications such as renal failure, acute respiratory discomfort syndrome (SDRA), heart failure, and coagulation abnormalities such as thrombosis [16]. The transmission occurs by person-to-person contact, via airborne droplets, or aerosols [3,13].

SARS-CoV-2 belongs to the Nidovirales order, Coronaviridae family, represented by four genera, such as α, β, δ, and γ-Coronaviruses [17,18]. The coronavirus is an enveloped single-strand RNA virus and non-segmented virus which has crown-shape glycoprotein spikes projecting from its surface. These glycoproteins attach to cellular receptors on the host cells and mediate viral entry, resulting in interspecies transmission and pathogenesis [19]. Cell entry of SARS-CoV-2 occurs through the binding of the spike glycoprotein (glycoprotein S) to angiotensin-converting enzyme (ACE2) expressed on the surface of the host cells, with the lung tissues being the main target [20]; therefore, intervention at the stage of adsorption/binding or replication of the virus using therapeutic agents can effectively block viral infection [21]; additionally, SARS-CoV-2 presents encoded non-structural proteins which participate in viral replication and pathogenesis [22,23]. One of these proteins, cysteine protease papain-like, is essential for the viral replication and it affects post-translational modifications on host proteins, contributing to the evasion of host immune responses [22,24].

RNA viruses are capable of infecting humans, and, when adapted, they can develop mutations, resulting in different variants, which has implications for the development of effective treatments [25]. Until now, five SARS-CoV-2 variants of concern for the World Health Organization (WHO) were identified: (i) α (B.1.1.7), in 2020 in the United Kingdom (UK); (ii) β (B.1.351), in 2020 in South Africa; (iii) Δ (B.1.617.2), in 2020 in India; (iv) γ (P.1), in 2021 in Brazil; and (v) Omicron (B.1.1.529), in 2021 in South Africa; but its subvariants BA.1, BA.2, BA.3, BA.4, and BA.5 were also identified elsewhere [25].

**Figure 1:**
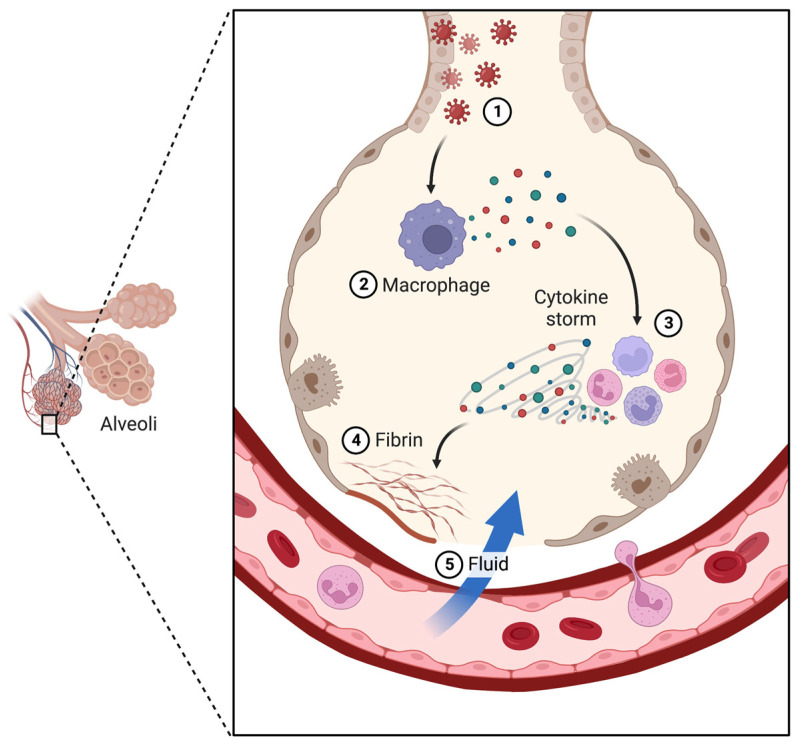
Cytokine storm. (**1**) Coronavirus infects lung cells. (**2**) The immune cells recognize the virus, are activated, and produce cytokines. (**3**) Cytokines attract more immune cells, which, in turn, produce more cytokines, creating a cycle of inflammation that damages the lung cells, resulting in (**4**) fibrin formation. (**5**) Fluid fills the lung cavities, leading to respiratory failure. Figure created with BioRender.com.

Patients infected with COVID-19 present high serum levels of inflammatory cytokines, such as IL-6, TNF-α, IL-1β, IL-8, and cytotoxic peptides (e.g., granulysin and perforin), resulting in a mechanism appropriately named “cytokine storm” [26]. The cytokine storm is a life-threatening systemic inflammatory syndrome involving elevated levels of circulating cytokines and immune-cell hyperactivation that can be triggered by various therapies, pathogens, cancer, autoimmune conditions, and COVID-19, generating exacerbated lung damage [27]; thus, cytokine storm implies that the levels of released cytokines are injurious to host cells (Figure 1); however, defining clinical criteria for the so-called cytokine storm is challenging, and studies propose a series of features such as clinical symptoms and laboratory findings to confirm the status [28,29]. Moreover, the proposition that the cytokine storm is pathological has also been met with skepticism [30]. In addition to this inflammatory picture, as lymphocytes are directly invaded by SARS-CoV-2 virus or indirectly damaged by the cytokines, lymphocytopenia usually is a prominent marker of COVID-19 [31].

The National Institute of Health (NIH) classified COVID-19 according to clinical symptoms, hemodynamics, organ function, laboratory, and radiographic abnormalities. The classification is (i) asymptomatic; (ii) mild; (iii) moderate; (iv) severe; and (v) critical illness. In several cases, the classification is difficult since it varies a lot, including lack of clinical manifestation until the presentation of acute respiratory distress syndrome, multiple organ dysfunction, and septic shock [25].

For the detection and confirmation of COVID-19 diagnosis, besides the clinical signs and patient history, diagnostic tests are mainly required, such as molecular tests (real-time polymerase chain reaction (PCR)), serological tests, and image analysis (computed tomography, X-ray of chest, or ultrasound of the lungs) [25].

## 3. Available Treatments for COVID-19

Due to the pandemic situation, the FDA has issued emergency-use authorization (EUA) for several medicines that were still undergoing clinical trials, such as anti-inflammatory and antiviral drugs, immunomodulator agents, and anti-SARS-CoV-2 monoclonal antibodies [25,32]; nevertheless, clinical studies regarding these treatments have shown good and bad results regarding effectiveness as well as their capacity to neutralize certain coronavirus strains in vitro [25].

The clinical usage of COVID-19 treatments is very complex and depends on the severity of the illness and risk factors. The COVID-19 clinical course occurs in two phases: (1) an early phase in which the replication of SARS-CoV-2 is more expressive before or right after the symptoms (in this situation, antiviral medications and antibody-based treatments are shown to be more effective); and (2) a later phase, driven by the release of cytokines and the activation of coagulation system, in which there are prominent hyperinflammatory and prothrombotic activities. In the late phase, anti-inflammatory drugs, such as corticosteroids and immunomodulating therapies, or even a combination of these therapies, can help diminish the hyperinflammatory state [33].

Among the antiviral therapies explored for COVID-19, Ivermectin figured in the early pandemic. Although the referred drug is known as an efficient antiparasitic drug, it was demonstrated that the drug could also inhibit SARS-CoV-2 replication in vitro [34]; however, the drug did not reduce the risk of developing severe COVID-19 and it is not indicated nowadays for patients [25]. Chloroquine and hydroxychloroquine were also the focus of tremendous public attention [35]; however, the results have failed to show survival benefit with these drugs, or even that they prevented SARS-CoV-2 infection or symptomatic COVID-19 illness [25,36,37,38,39]; moreover, chloroquine and hydroxychloroquine present wide-ranging drug interactions and potential cardiotoxicity [35]. Lopinavir/ritonavir were also considered to be used for COVID-19 therapy, since they are drugs used to treat HIV, but they also did not show any benefit to COVID-19 patients [25,40]. Other medications can be used for COVID-19 patients, such as molnupiravir (which reduced the hospitalizations and deaths of patients with mild COVID-19 and on nonvaccinated people [41,42]), and paxlovid (which reduced hospitalizations and deaths of patients when it was used within three days of symptoms appearing [43], and which was indicated for mild and moderate COVID-19 patients) [25]. Although remdesivir can also be a drug option, its therapeutical use is quite controversial because it did not show good results in terms of length of stay, initiation of oxygen therapy, or mortality [37]; however, other studies have shown that remdesivir was able to reduce the recovery time and death of patients with COVID-19 [44,45,46,47].

As regarding the roles of neutralizing antibodies targeting SARS-CoV-2, these have been extensively studied in ongoing clinical trials. One of them is convalescent plasma therapy, approved by the FDA for patients with severe life-threatening COVID-19 [48]. Although it appeared promising, multiple studies evaluating this therapy have generated mixed results. An example is a retrospective study based on a U.S. national registry report that patients hospitalized with COVID-19, not under mechanical ventilation, receiving a transfusion of convalescent plasma containing higher anti-SARS-CoV-2 IgG antibody, had a risk of death that was lower than patients who received a transfusion of convalescent plasma with lower levels of antibody [49].

Monoclonal antibodies (mAb) have been considered the most promising treatments for COVID-19. The REGN-COV2 is a key example of mAb, which contains two noncompeting IgG1 antibodies (casirivimab and imdevimab) targeting RBD on the SARS-CoV-2 spike protein. Preliminary data from a Phase 3 trial of REGN-COV revealed a 70% reduction in hospitalization or death in non-hospitalized COVID-19 patients [50].

Since SARS-CoV-2 and SARS-CoV share similarities, studies suggest the use of SARS antiviral monoclonal antibodies in patients with SARS-CoV-2. Many monoclonal antibodies have been described to identify the S1 fragment of SARS-CoV and RBD in subunit S1. This is the most important goal for SARS-CoV-2 [51] because monoclonal antibodies can block the interaction of RBD and its ACE2 receptor [52]. There are monoclonal antibodies binding the epitopes in unit S2 of SARS-CoV, suggesting neutralization [53]. So far, there are 10 monoclonal antibodies targeting S1 fragment of SARS-CoV and, 4 targeting S2 fragment of SARS-CoV [54].

Researchers have also studied other neutralizing antibodies that block COVID-19. One example is 47D11, which was discovered using an ELISA-(cross) reactivity approach to assess antibodies contained in supernatant samples from immunized transgenic mice. These antibodies demonstrated that they bind to SARS-CoV-2 and could strongly inhibit the virus infection on Vero cells [55]. There are also reports on four human-origin monoclonal antibodies (B5, B38, H2, and H4) from convalescent patients which demonstrated that they could bind to RBD. The ability of each antibody to inhibit binding between RBD and ACE was evaluated and showed that B38 and H4 have complete competition with ACE2 for binding RBD, while, in contrast, B5 displayed partial competition and H2 demonstrated no competition with ACE2 for RBD binding [56].

Immunomodulating agents have also been explored for COVID-19 treatment, such as corticosteroids, IFN-β-1a, IL-1 antagonists, anti-IL-6 receptor (tocilizumab, sarilumab, and siltuximab), Janus kinase (JAK) inhibitors (baricitinib, ruxolitinib, and tofacitinib), and tyrosine kinase inhibitors (acalabrutinib, ibrutinib, and rilzabrutinib) [25].

In addition to the pharmacological treatment, the complementary therapy must be conducted on COVID-19 patients to improve the symptoms and oxygen saturation (oxygenation and ventilation) [25].

Besides all the above therapies and others still in research phase, vaccination is the most effective way to avoid coronavirus infection. Currently, some vaccines were granted authorization in the USA: BNT162b2 vaccine (mRNA-based, BioNTech/Pfizer, New York, NY, USA) [57], mRNA-1273 vaccine (mRNA based, Moderna, Cambridge, MA, USA) [58], and Ad26.COV2.S vaccine (Janssen Research and Development, Beerse, Belgium) [59]. ChAdOx1 nCoV-19 vaccine (AstraZeneca, Cambridge, UK) [60] has been authorized for emergency use in several countries, but has not been granted an EUA from the FDA [25], while NVX-CoV2373 vaccine (Novavax, Gaithersburg, MD, EUA) had clinical trials performed [61,62]. Other vaccines had their emergency use approved or were approved as prevention worldwide, such as CoronaVac (Sinovac Biotech, Haidian District, Beijing, China), Covaxin (Bharat Biotech, Hyderabad, Telangana, India), and Sputnik V (Gamaleya Research Institute of Epidemiology and Microbiology, Moscow, Russia) [25].

## 4. Crosstalk of Animal-Derived Toxins and COVID-19

Animal venoms are rich in active biological compounds [63] and several toxins from different venomous and poisonous animals, whether aquatic or terrestrial, have already had their antiviral potential determined against many types of viruses. For dengue virus, a PLA_2_ from *Bothrops leucurus* snake venom was able to decrease amounts of viral RNA [64]; similarly, a PLA_2_ from honey bee *Apis mellifera* also prevented intracellular release of the viral capsid protein of human immunodeficiency virus (HIV), and hepatitis C virus (HCV), among others, suggesting it blocks viral entry into cells [1,65]. In 2011, Li et al. observed that the optimized toxin mucroporin from *Lychas mucronatus* scorpion venom, mucroporin-M1, was able to perform a potent antiviral activity against measles, influenza H5N1, and SARS-CoV viruses, demonstrating that toxins could be prototypes of new antiviral drugs [66].

As regards animal toxins’ effects on SARS-CoV-2, there are still only a few studies which give some evidence of antiviral activities against COVID-19 (Table 1). In a recent study, researchers explored a few PLA_2_ and their subunits—two PLA_2_ from the venom of the krait *Bungarus fasciatus* (BF-PLA2 I and II), one from *Viper ursinii renardi* (Vur-PLA2), and one from *Viper nikolskii* (HDP-1 and HDP-2). The antiviral activity of the *Viper nikolskii-*derived molecules (HDP-1 and HDP-2—dimeric proteins; HDP-1I and HDP-2P—subunits) were tested through the cytopathic effects (CPE) of SARS-CoV-2 on Vero E6 cells, a cell line commonly used in virology because viruses produce CPE [67]. The results showed that all PLA_2_ demonstrated antiviral activity and prevented morphological changes with HDP-1 and HDP-2, presenting the most potent antiviral activity and inhibiting close to 50% of the CPE. The authors speculated that this potent antiviral activity could be due to the enzyme phospholipolytic activity being responsible for the destruction of the viral envelope. On the other hand, Vur-PLA_2_ was less potent, while BF-PLA_2_-I and II showed the lowest antiviral activity, inhibiting less than 50% of the CPE [68].

Also from snake venoms, three peptide dimers derived from the C-terminus of the myotoxin bothropstoxin-I, from *Bothrops jararacussu*, were tested against SARS-CoV-2. They demonstrated inhibition of viral infection, targeting the viral papain-like cysteine protease with low and micromolar potency [69]; still, we can cite the Cobrotoxin, from the *Naja naja atra* snake, which could be a candidate for alternative therapy for COVID-19 because it may have an inhibitory role on the cytokine storm caused by SARS-CoV-2 in COVID-19 [70].

As regards bee venom (*Apis mellifera*), some studies have been published regarding its action on SARS-CoV-2. Authors hypothesized that the whole venom may attenuate the cytokine storm caused by SARS-CoV-2 and could be used in a prophylactic context for COVID-19 [71,72]; in addition, melittin was tested for in vitro assay using Vero cells and it was found that it could neutralize SARS-CoV-2 virus, showing more pronounced antiviral activity at 12 h with 95% of viral reduction; also, the effect of melittin on the ability of the virus to infect Vero cells was studied through high-throughput proteomic analysis. The omics analysis revealed that proteins were found to be down-regulated in the cells following melittin treatment, indicating that the toxin induces a metabolic effect and not merely viral lysis [73]. Although this study shows benefits of melittin against SARS-CoV-2, a formulation containing melittin must be very well adjusted so that it causes no cytolytic effects [73,74].

**Table 1:** Animal toxins targeting COVID-19.

Toxins	Species	Animal	Mechanism	Year	Ref.
Bee venom	*Apis* *mellifera*	Bee	Hypothesis: attenuate cytokine storm caused by SARS-CoV-2.	2020	[71,72]
			Prophylactic context for COVID-19.		
Melittin	*Apis* *mellifera*	Bee	In vitro assay using VERO cells: neutralizes the SARS-CoV-2 virus.	2022	[73]
Dermaseptin-S9	*Phyllomedusa sauvagii*	Frog	Inhibitor of SARS-CoV-2 spike glycoprotein by protein-peptide analysis in silico by docking.	2020	[75]
Meucin18 and its mutation	*Mesobuthus* *eupeus*	Scorpion	Inhibitor of SARS-CoV-2 spike glycoprotein. Protein-peptide analysis in silico by docking.	2021	[76]
Cobrotoxin	*Naja naja* *atra*	Snake	Inhibitory effect on the cytokine storm caused by SARS-CoV-2 in COVID-19.	2020	[70]
Dimeric peptides from BthTX-I (PLA2)	*Bothrops* *jararacussu*	Snake	Inhibitory activity against the Papain-like protease of SARS-CoV-2.	2021	[69]
PLA2	*Vipera* *nikolskii*	Snake	Inhibition of SARS-CoV-2 spike glycoprotein-mediated cell-cell infusion.	2021	[68]

Frogs’ venoms can be interesting for studying their roles for SARS-CoV-2. The Dermaseptin-S9 toxin, from *Phyllomedusa sauvagii*, was studied along with ACE2 and eight negative control molecules and the study shows their abilities to act as inhibitors of SARS-CoV-2 spike glycoprotein. These were assessed by protein–peptide analysis in silico by docking [75]; however, other studies must be conducted to lead to an increased affinity and specificity of Dermaseptin-S9 against SARS-CoV-2 [75].

Meucin18, from the *Mesobuthus eupeus* scorpion, had its binding ability evaluated by molecular docking and this showed that the toxin was able to prevent the ACE receptor binding with the SARS-CoV-2 spike protein [76]. In the same study, using in silico analysis, the authors reported that the mutated Meucin18 toxin (A9T) more effectively inhibited the ACE–spike interaction than native toxin [76].

Our in-depth searching using the main bibliographic databases reveal only seven studies even applying different match descriptors such as ‘COVID-19 and venoms’ or ‘COVID-19 and venom-derived toxins’; moreover, to the best of our knowledge, none of these toxins are under clinical trials for treating COVID-19.

## 5. Venom-Derived Toxins as COVID-19 Therapy: New Perspectives

**Figure 2:**
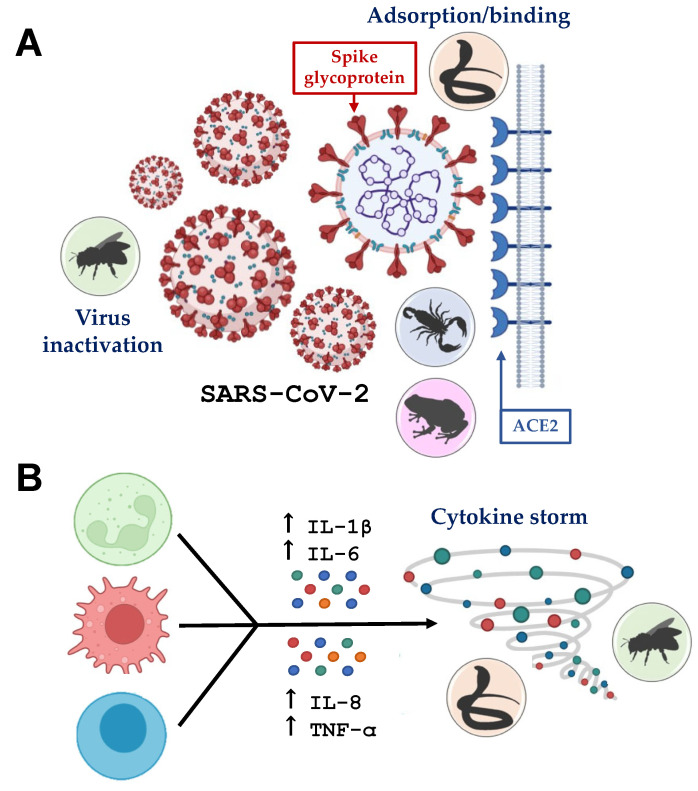
Targets of venom-derived toxins during SARS-CoV-2 infection. (**A**) Virus inactivation or absorption/binding inhibition. During SARS-CoV-2 infection, the enveloped virus binds its spike glycoprotein (red) to ACE2 receptor (blue) from host cells. (**B**) Inhibition of cytokine storm. Neutrophils (green), macrophages (red), and T cells (blue) are activated and release pro-inflammatory cytokines, triggering a cytokine storm. The animals (bee, snake, scorpion, and frog) are placed according to their possibly therapeutic action. Figure created with BioRender.com.

Vaccines and antiviral drugs are effective in fighting viral replication in host cells. Although highly advantageous, the development of vaccines and antiviral drugs follows high standards of demand and procedural protocols in all stages of the test (in vitro, in vivo, and clinical trials) and their development needs to succeed in many phases until registration and commercialization, which take a long period in addition to high costs. As an exception, the COVID-19 pandemic demonstrated a different experience through the rapid production of vaccines, along with accountable concomitant studies trying to discover novel compounds with SARS-CoV-2 antiviral activity. Such activity has been investigated through approaches such as in silico and in vitro studies with different effects on the phases of virus pathogenesis and immunostimulant effects [77]. Notably, venom-derived molecules have been evaluated as potential candidates for the development of novel antivirals (Table 1) [68,69,70,71,72,73,75,76]. The animals, virus targets, and potential mechanisms are represented in the Figure 2.

The dynamic of venom-induced biological activities in humans (e.g., antiviral, anti-inflammatory, and coagulopathies) cause venom compounds to be a rich resource for possible design of new drugs targeting COVID-19, especially those provided from snake venoms. Although a promising future is foreseen for venom-derived drugs, unfortunately, few studies focus on this [78]. For instance, there is a direct relationship of venom-derived toxins and the systems renin-angiotensin and kinin-kallikrein, with direct action on angiotensin-converting enzyme 2 (ACE2), and it is well known that SARS-COV-2 down-regulates ACE2, which significantly contributes to the pathophysiology of COVID-19 [71,79]; moreover, developing antithrombotic agents derived from snake venoms could be promising to prevent and treat cardiovascular disorders in COVID-19 patients since the clinical management of COVID-19-associated thrombosis is very complex, producing many challenges related to the use, dose, and choice of anticoagulants [80]; indeed, blood circulation, particularly thrombosis and haemostasis, is one of the major targets of several snake venom proteins [81].

Venom-derived anti-inflammatory toxins could also be potential coadjutant treatments for COVID-19.

A metalloproteinase from the *Bothrops moojeni* snake venom, called BmooMP-alpha-I, was shown to be able to inhibit TNF-a through its degradation, in both in vitro and in vivo assays [82].

The synthetic peptide HsTx2 was demonstrated to reduce TNF-α, IFN-γ, and IL-6 levels in BALB/c mice [83]. This molecule is based on peptide HsTx2 from the *Heterometrus spinifer* scorpion venom, and it presented neuroprotective effects in rats during ischemic stroke [84].

A low dose of honeybee venom was also able to reduce IL-1β, IL-6, and TNF-α levels during in vivo assays and, using higher doses, these levels might be improved [85].

*Naja naja atra* snake venom and its toxin neurotoxin-Nna has been shown to decrease IL-1β and TNF-α levels in the kidney and the serum of rats, respectively [86,87]; in addition, *N. n. atra* venom can inhibit IL-6 and TNF-α production in systemic lupus erythematosus in mice [88].

Based on the foregoing, anti-inflammatory toxins are seen to be able to affect the human immune system [89] and could be used as a coadjutant therapy to COVID-19 in special targeting of the cytokine storm; therefore, bioprospecting novel therapeutic drugs derived from venoms for COVID-19 have demonstrated several advantages. Among the matters discussed above, we can also highlight that some venom-derived peptides can present low immunogenicity and can be easily optimized and manufactured in the laboratory; however, these toxins still do not have acceptable efficacy and do not demonstrate the risk/benefit ratio for human treatments, and appropriate clinical studies are needed for their use; thus, more studies in the toxinology field should be developed to suggest new antiviral drugs against COVID-19. Bioinformatics tools (i.e., venomics) could also facilitate this.

## 6. Conclusions

After more than two years since the COVID-19 outbreak, there is no specific therapy for this life-threating disease, making the search for an effective therapy uniquely importance to the world at present; thus, bioprospecting antiviral drugs in venoms is very promising since several venom-derived compounds have demonstrated that they can be effective in similar biological systems as those affected by SARS-CoV-2 (e.g., coagulation, hemodynamics, and immune and renal systems). Although there are just a few studies in the scientific literature, the venom-derived drugs currently in development, and the recent gains in knowledge of the virus and the disease itself, give us hope for finding new therapies for COVID-19 soon.

## Data Availability

Not applicable.
